# A qualitative study assessing the acceptability of a multi-agent AI Chatbot for providing HIV and mental health support among men who have sex with men and transgender women in KwaZulu-Natal, South Africa

**DOI:** 10.1093/trstmh/traf143

**Published:** 2026-01-13

**Authors:** Hilton Humphries, Lindani Msimango, Zimasa Tshawe, Natasha Gcelu, Kurt Ferreira, Jacqueline Pienaar, Elise M van der Elst, Danielle Giovenco, Don Operario, Eduard J Sanders, Alastair van Heerden

**Affiliations:** Centre for Community-Based Research, Human Science Research Council, Sweetwaters 3201, South Africa; Division of Social and Behavioural Sciences, School of Public Health, Faculty of Health Sciences, University of Cape Town, Cape Town 7701, South Africa; Department of Psychology, University of KwaZulu-Natal, Pietermaritzburg 3201, South Africa; Centre for Community-Based Research, Human Science Research Council, Sweetwaters 3201, South Africa; Centre for Community-Based Research, Human Science Research Council, Sweetwaters 3201, South Africa; Centre for Community-Based Research, Human Science Research Council, Sweetwaters 3201, South Africa; Worien, Hilton 3245, South Africa; Aurum Institute, Johannesburg 2193, South Africa; Faculty of Social and Behavioural Sciences, University Utrecht, Utrecht 3508, The Netherlands; Hubert Department of Global Health, Rollins School of Public Health, Emory University, Atlanta, GA 30322, USA; Department of Behavioral, Social, and Health Education Sciences, Emory University, Atlanta, GA 30322, USA; Aurum Institute, Johannesburg 2193, South Africa; Nuffield Department of Medicine, Oxford University, Oxford OX3 7BN, UK; Syndicate for Public Science and Emerging Technologies, Wits Health Consortium, Johannesburg 2193, South Africa

**Keywords:** artificial intelligence, chatbots, HIV prevention, HIV treatment, key populations, mental health, MSM, South Africa, transgender women

## Abstract

**Background:**

Transgender women (TGW) and men who have sex with men (MSM) are disproportionately affected by human immunodeficiency virus (HIV) and mental health challenges. Mental well-being influences uptake and adherence to HIV prevention and treatment. However, gaps in mental health service delivery present challenges for scalability in public health systems. Artificial intelligence (AI)-driven chatbots may offer a novel, scalable solution to expand access to mental health support.

**Methods:**

This qualitative study was conducted at the Aurum POP INN clinic in Pietermaritzburg, KwaZulu-Natal. A multi-agent AI chatbot, designed to simulate supportive counselling based on the Inuka model, was piloted with TGW and MSM. Ten participants engaged in in-depth interviews after interacting with the chatbot. An additional 34 participants experienced both chatbot and in-person counselling through a randomised crossover design and then participated in four focus group discussions. The Unified Theory of Acceptance and Use of Technology and the Acceptability of Healthcare Interventions Framework guided the analysis.

**Results:**

The chatbot was generally acceptable, with participants valuing its privacy, convenience and human-like interaction. Acceptability was enhanced by associations with modernity and anonymity. Trust, usability and accessibility improved engagement. Key barriers included slow response times, limited rapport and repetitive messaging.

**Conclusions:**

AI chatbots offer a promising, scalable approach to supporting mental health among key populations in HIV care.

## Introduction

The use of large language model (LLM)-driven chatbots in public health interventions is gaining momentum, offering scalable, low-cost solutions to some of the most persistent challenges in global health. These chatbots show promise for improving access, engagement and responsiveness in low-resource settings, yet their use in emotionally complex domains like mental health remains underexplored.^1–3^ In particular, their role in providing mental health support to vulnerable and hard-to-reach key populations—like men who have sex with men (MSM) and transgender women (TGW)—is an area requiring exploration, especially considering the disproportionately high human immunodeficiency virus (HIV) risk and significant mental health burdens experienced by these groups.^4,5^

MSM and TGW are the fastest growing HIV risk populations in South Africa, the country with the world’s largest HIV epidemic.^4–6^ In a systematic review and meta-analysis for trends in HIV testing and care cascade outcomes, the pooled estimate for MSM living with HIV and aware of their status was 56% and those currently on antiretroviral therapy (ART) was 73% in southern Africa in 2020.^4^ Treatment cascade indicators for TGW in three cities in South Africa were reported in a recent study in 2023; in Johannesburg, an estimated 54.2% (95% confidence interval [CI] 45.8–62.4) of transgender women with HIV knew their positive status, in Cape Town this was 24.2% (95% CI 15.4–35.8) and in Buffalo City this was 39.5% (95% CI 27.1–53.4).^4^ Among those who knew their status, 82.1% (95% CI 73.3–88.5) in Johannesburg, 78.2% (95% CI 57.9–90.3) in Cape Town and 64.7% (95% CI 45.2–80.2) in Buffalo City were on ART. Of those on ART, 34.4% (95% CI 27.2–42.4) in Johannesburg, 41.2% (95% CI 30.7–52.6) in Cape Town and 55.0% (95% CI 40.7–68.4) in Buffalo City were virally suppressed.^4^

Intersecting with a disproportionately high HIV incidence, MSM and TGW also face a substantial burden of mental health challenges. Common mental health problems—including depression and anxiety—are prevalent among MSM and TGW in South Africa due to social factors including exposure to anti-MSM stigma, internalised stigma, concealment of sexual identity, isolation and loneliness.^7–9^ Unaddressed mental health problems among MSM and TGW contribute to challenges across the HIV prevention and care continua, including poor adherence to daily oral pre-exposure prophylaxis (PrEP) and ART.^8,9^ Unfortunately, few specific programmatic efforts have been devoted to improving mental health as part of initiatives aimed at ending HIV in Africa, and mental health support services remain poorly accessible to most people.^8^ Given the severe shortage of mental health providers in South Africa and other low- and middle-income countries, LLM-driven chatbots could offer an innovative path towards closing the mental health treatment gap.^10–12^ Recent data show the potential of chatbots in the mental health space—with users responding positively to the personalised, non-judgemental, human-like interactions and accessible nature of these interventions. However, poor performance issues may hinder engagement and ongoing use of chatbots, and there remain concerns about a chatbots ability to identify crises requiring urgent attention.^1–3,10–14^ Research on chatbot acceptability in low-resource settings is needed to understand how they are experienced and could address the needs of end-users in these settings where mental health services remain insufficient.

To address this gap, we piloted the use of an LLM-driven chatbot to deliver a digital adaptation of the Friendship Bench (FB) problem-solving therapy model—a community-based intervention originally developed in Zimbabwe and successfully implemented in multiple low-resource settings.^15–18^ The chatbot provided introductory mental health support and was offered to MSM and TGW using HIV prevention or treatment programs at the Aurum Institute’s POP INN clinics as part of a broader mental health well-being initiative.^7^ This exploratory study sought to understand what factors would affect the acceptability of and willingness to use a mental health support chatbot, highlighting opportunities to maximise the benefits of digital health interventions for underserved and high-need users. Through qualitative exploration, we offer user-centred insights into how these technologies may enhance mental health support among MSM and TGW.

## Methods

### Study community

The study was conducted at the Aurum POP INN clinic in Pietermaritzburg, KwaZulu-Natal, South Africa. Pietermaritzburg is the capital city of the KwaZulu-Natal province and has a population of approximately 900 000 people. The city is situated within the uMgungundlovu district, which has a high HIV burden, having an antenatal HIV prevalence of 40.7%.^19^ There are limited options available for members of the lesbian, gay, bisexual, transgender, queer/questioning, intersex community to access services focused on their health needs and, where available, they are concentrated in urban centres, making access difficult for those in outlying areas.

### Chatbot development

This study includes data from a qualitative study investigating the factors that influence the acceptability and usability of a chatbot for providing mental health support to MSM and TGW. The multi-agent chatbot utilised a novel architecture where each message was processed by five agents, each employing a distinct epistemological framework (e.g. rapport building, psychoanalysis, cognitive behavioural therapy, engineering first principals). A coordinating agent synthesised these responses and applied an appropriate stylistic tone tailored to the target population before delivering the final message. The agents all leveraged gpt-4-turbo via the OpenAI API. The chatbot was developed to provide an introductory module of the Inuka coaching training program. Inuka coaching is an online problem-solving therapy training program based on the evidence-based FB program.^15–18^

The FB program is an intervention designed to target symptoms of common mental health problems (depression, anxiety). In ongoing work by the Aurum Institute, an FB-based coaching intervention has been adapted to address cascade of care outcomes including PrEP medication adherence, ART adherence and viral suppression. The FB program showed that task-shifting of mental health care to lay health workers improved mental health outcomes when compared with the standard of care.^12^ The core components of the FB approach to problem-solving therapy include identifying the problem; exploring the individual’s experience and manifestations of the problem; brainstorming feasible solutions and options available; setting a specific, measurable, achievable and realistic action plan; implementing the action plan; and providing reassurance and follow-up. The chatbot was trained using anonymous transcripts from previous Inuka in-person online coaching sessions (data collected in the AUR6-8-403: mental health pilot study at Aurum). We used the Telegram mobile app as an interface to the LLM chain that leveraged the OpenAI gpt-4-turbo model as the foundation on which the agents operated and were trained on for this study.

### Procedures

**Figure 1.**
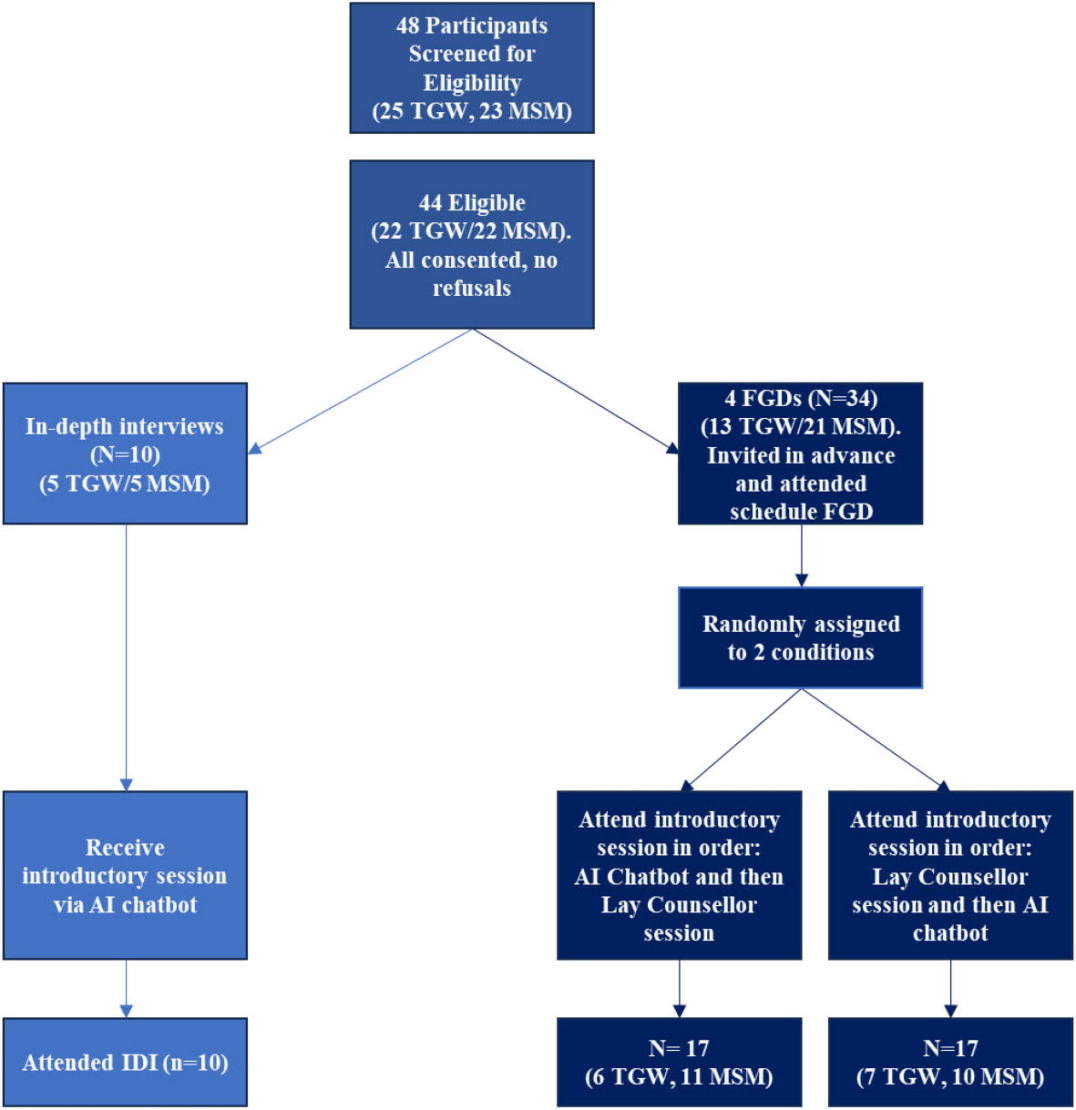
MSM and TGW study flow overview.

The study included a purposive sample of HIV status-neutral MSM and TGW ages 18–29 years who screened positive for mild to moderate symptoms of depression using the nine-item Patient Health Questionnaire (PHQ-9). Using the introductory module of the Inuka counselling approach, the AI chatbot was trained to provide introductory, basic mental health support. Participants accessed the chatbot using a study-provided laptop. They were trained to use the chatbot by the interviewer and then allowed to engage with the chatbot for 20–30 min. The facilitator was present to help if needed during the session. After participants had finished their session with the chatbot, they were given a comfort break and then invited to start the interview. In-depth interviews (IDIs) were completed with 10 TGW and MSM to get feedback on the interaction with the chatbot; an additional 34 TGW and MSM received chatbot counselling and in-person counselling using a randomised crossover design (Figure 1) (receiving introductory chatbot mental health counselling and then in-person counselling or vice versa) after which they participated in a focus group discussion (FGD) (n=4). Finally, one FGD was held with three lay mental health coaches certified in the FB counselling method. This article focuses on data from the MSM and TGW data collection activities only. The study was conducted between August 2024 and October 2024. IDIs and FGDs explored the acceptability, usability, utility and user experience of the different counselling approaches.

The Unified Theory of Acceptance and Use of Technology (UTAUT)^20^ and the Acceptability of Healthcare Interventions Framework (AHIF)^21^ frameworks were used to conceptualise and understand important facets of acceptability among users. The UTAUT identifies key factors that influence technology adoption, including performance expectancy (the belief that the technology will be useful), effort expectancy (ease of use), social influence and facilitating conditions (the resources and support available). In contrast, the AHIF is used to assess how acceptable a health intervention is to those delivering or receiving it.^2^ It includes constructs such as affective attitude (how someone feels about the intervention), burden (perceived effort), ethicality, intervention coherence (understanding of the intervention) and self-efficacy (confidence in using it).

The IDIs and FGDs included a non-probability convenience sample of HIV status-neutral MSM and TGW recruited from participants at the POP INN clinic in Pietermaritzburg. Recruitment was done by research assistants at the POP INN clinic and was focused on MSM and TGW who had used or were using services from the POP INN clinic. All participants had been or were clients of the clinic, some of whom had previously accessed basic psychosocial support through the clinic, although none were actively engaged in counselling at the time of recruitment. Participants were purposefully selected from the population of clients attending the Pietermaritzburg POP INN clinic for HIV or PrEP services. Eligible participants were MSM or TGW who were initiating, using or receiving a refill of ART or PrEP and where mental health screening was conducted as part of routine clinic visits. Those who met the eligibility criteria were subsequently invited to participate in the study.

The IDI guides were developed by reviewing current literature and engaging in discussions with the research team, local community partners, thematic experts and young people, as well as being informed by the Unified Theory of Acceptance and Use of Technology and the Acceptability of Healthcare Interventions Framework to ensure theoretical rigour. Participants completed IDIs and FGDs when convenient and in their preferred language (English or isiZulu). All interviews and FGDs were completed by experienced, trained interviewers proficient in both English and isiZulu. To avoid disruptions, interviews and FGDs were conducted in a private room and lasted 60–90 min. Interviews were audio recorded, transcribed and then translated into English. The transcripts were quality checked against the audio recordings for accuracy by a research assistant. Participants were reimbursed 160.00 South African rands for their time, inconvenience and travel.

### Reflexivity statement

The research team acknowledged that our identities, professional backgrounds and experiences could influence data collection and interpretation. The data collectors were cis-gender African researchers with advanced degrees, working with MSM and TGW who accessed services through a non-governmental partner who formed part of the larger research team. Differences in education, socio-economic status and gender identity may have influenced participants’ comfort and openness during interviews, while shared cultural or racial backgrounds could have facilitated rapport. The team engaged in ongoing reflexive discussions to consider how our positionalities, prior experiences with HIV and mental health research and professional roles might shape both the questions asked and the interpretation of participants’ narratives.

### Data analysis

The analysis was guided by two complementary frameworks (the UTAUT and the AHIF).^20,21^ Together, these frameworks offer a comprehensive view of both the psychological and contextual factors shaping acceptance and use of new health technologies or services. The analysis also focused on identifying key recommendations to improve usability and acceptability to inform future development of the chatbot. Through multiple independent readings (HH, ZT, LM, LL and NG) of a subset of transcripts, themes and codes were identified and reviewed until consensus about the representativeness of the theme was reached by the primary research team of this qualitative study. The themes were documented following guidelines by Braun and Clarke.^22^ The code book was then applied to the full dataset iteratively to identify any new themes that needed to be added. Final coding was facilitated using NVivo-12 (Lumivero, Denver, CO, USA). This final coding was done independently by four members of the research team (ZT, LL, LM and NG), followed by group meetings to reach consensus and saturation on themes and codes and continued improvements on interrater reliability and adaptations to the codebook. Once interrater testing was complete, the themes were used as a basis for further analysis and discussion of the overall research question.

## Results

A total sample of 44 participants were enrolled in the study. This included 21 cis-gendered men who identified as MSM and 23 TGW. The age range of participants in the study was 18–29 y. In total, we completed 10 IDIs and 4 FGDs. Key demographic information is provided in Table 1. Participants had varying levels of psychosocial support that they could access, with the majority reporting access to a little or some psychosocial support (Table 2). All participants reported prior use of social media platforms, suggesting they may have a general familiarity and comfort with digital technologies.

**Table 1.** Participant’s demographics (n=44).

Characteristics	Category	Values
Age (years), median (IQR)		23 (20–25)
Gender, % (n)	TGW	52 (23)
	Cis-gender male (MSM)	48 (21)
Education, % (n)	High school (grade)	50 (22)
	Tertiary/vocational	34 (15)
	High school (grade 12)	11 (5)
	High school (grade 10)	5 (2)
Main activity, % (n)	Unemployed	30 (13)
	Student/learner	27 (12)
	Informally employed	20 (9)
	Formally employed	18 (8)
	Tertiary/vocational	2 (1)
	Choose not to work	2 (1)
Relationship status, % (n)	Not in a relationship	45 (20)
	Having a partner but living separately	31.8 (14)
	Once off sex partners	11 (5)
	Married, living together	5 (2)
	Living with your partner	5 (2)
	Married, living separately	2 (1)
Relationship length, % (n)	Long term (>6 months)	27.3 (12)
	Short term (≤3 months)	9.1 (4)
	Medium term (3–6 months)	6.8 (3)
	Long term (>6 months)	27.3 (12)
Multiple sexual partners, % (n)	No	56.8 (25)
	Yes	43.2 (19)
HIV status (self-reported), % (n)	Negative	52 (23)
	Positive	48 (21)
Currently using PrEP or ART, % (n)	Yes	100 (44)
	No	0 (0)
Sexual and reproductive health services used, % (n)	Yes	100 (44)
	No	0 (0)
PHQ-9 classification (score), % (n)	Mild depression (5–9)	52 (23)
	Moderate depression (10–14)	48 (21)

**Table 2.** Emotional and informational support (n=44).

Statement	None of the time, % (n)	A little of the time, % (n)	Some of the time, % (n)	Most of the time, % (n)	All of the time, % (n)
How often is there someone to confide in or talk to about yourself or your problems?	9.1 (4)	20.5 (9)	31.8 (14)	15.9 (7)	22.7 (10)
How often is there someone to give you good advice about a crisis?	18.2 (8)	9.1 (4)	29.5 (13)	27.3 (12)	15.9 (7)
How often is there someone to give you information to help you understand a situation?	15.9 (7)	25.0 (11)	15.9 (7)	36.4 (16)	6.8 (3)
How often is there someone to share your most private worries and fears with?	25.0 (11)	22.7 (10)	22.7 (10)	15.9 (7)	13.6 (6)
How often is there someone to turn to for suggestions about how to deal with a personal problem?	11.4 (5)	22.7 (10)	31.8 (14)	18.2 (8)	15.9 (7)
How often is there someone who understands your problems?	13.6 (6)	29.5 (13)	25.0 (11)	15.9 (7)	15.9 (7)
How often is there someone whose advice you really want?	22.7 (10)	20.5 (9)	18.2 (8)	25.0 (11)	13.6 (6)
How often is there someone you can count on to listen to you when you need to talk?	13.6 (6)	18.2 (8)	27.3 (12)	13.6 (6)	27.3 (12)

Under the umbrella of three overarching thematic areas (benefits, challenges and additional features), we identified eight overall themes from the qualitative analysis: 1) benefits of the chatbot: convenience and accessibility, affective attitudes, privacy and anonymity and trustworthiness; 2) challenges of the chatbot: relationship building, poor performance, data privacy concerns and cost; and 3) suggested improvements to the chatbot. These themes reflect participants’ perceptions of the chatbot’s ease of use, emotional resonance, trustworthiness and fit within their daily lives. These findings each align with key constructs of the UTAUT2 and the TFA (Table 3).

**Table 3.** Alignment of key findings with guiding theoretical frameworks.

Theme	UTAUT/UTAUT2 constructs	TFA constructs	Interpretation
Convenience and accessibility	Performance expectancy—belief that the chatbot improves access to mental health support Effort expectancy—convenient to use, low effort Facilitating conditions—enabled by phone access and flexible timing for use	Burden—reduces logistical and emotional effort Perceived effectiveness—immediate response seen as helpful Opportunity cost—minimal disruption to daily life	Convenience and low effort drive adoption by fitting seamlessly into users’ routines
Affective attitudes: subthemes: enjoyment, comfort, emotional connection and modern and advanced mode of service delivery	Hedonic motivation—fun, friendly, enjoyable Social influence—sense of being modern or advanced Performance expectancy—positive feelings reinforce usefulness	Affective attitude—emotional responses toward the chatbot Intervention coherence—discomfort with robotic tone indicates misalignment with expectations	Positive emotional experiences increase motivation to use; lack of empathy reduces engagement and acceptability
Privacy and anonymity	Trust—belief that the chatbot protects confidentiality Facilitating conditions—secure design supports use Effort expectancy—privacy reduces disclosure concerns	Ethicality—aligns with moral values around confidentiality Self-efficacy—anonymity increases confidence to disclose Burden—reduces stigma-related burden	Perceived privacy strengthens trust and ethical acceptability, encouraging open engagement
Trust	Performance expectancy—belief in accuracy and quality of information Trust—confidence in reliability and organisational affiliation Facilitating conditions—legitimacy supports use	Perceived effectiveness—trust reinforces belief in benefit Ethicality—data security aligns with moral expectations Intervention coherence—uncertainty about ‘qualification’ to provide mental health support reduces fit	Trust is foundational for perceived usefulness and ethical acceptance; uncertainty undermines engagement
Relationship building	Hedonic motivation—lack of emotional resonance reduces satisfaction Social influence—preference for human connection	Affective attitude—low emotional connection reduces acceptability Intervention coherence—mismatch between expectations of counsellor versus chatbot support	Weak social presence limits engagement: emotional empathy could improve acceptability
Poor performance	Performance expectancy—repetitive or slow responses reduce usefulness Effort expectancy—poor usability increases effort	Burden—frustration adds effort Perceived effectiveness—poor response quality lowers benefit	Technical issues undermine usability and acceptability
Data privacy	Trust—uncertainty about data handling reduces confidence Facilitating conditions—security concerns reduce acceptability	Ethicality—fear of data misuse conflicts with moral expectations Self-efficacy—doubt in safe use reduces confidence	Mixed feelings about data privacy show acceptability depends on clear protection and transparency
Cost to use	Price value—unfavourable cost-benefit of data use Facilitating conditions—data cost as a structural barrier	Burden—financial strain Opportunity cost—limits access for low-income users	High data costs undermine otherwise positive perceptions of usefulness
Suggested improvements (features and design) (Table 4)	Facilitating conditions—broader platform availability and data-free access Performance expectancy—expanded features improve utility Hedonic motivation—personalisation and interactive design improve enjoyment	Self-efficacy—language and personalisation increase confidence and desire to use the chatbot Ethicality—ability to delete data aligns with moral expectations Burden—improved usability reduces effort	Enhancements that improve usability, personalisation, and data safety increase both acceptance and sustained engagement

### Benefits, challenges and additional features to enhance acceptability of using the chatbot

Overall, acceptability of the chatbot was good and was perceived by all participants as a valuable addition to mental health support services. Most participants felt that the chatbot was not a replacement for in-person mental health services but rather an addition to existing services and a valuable tool, especially at the early stages of facing mental health challenges or as a way of receiving ongoing support. The factors that mediated acceptability are presented under three main categories: benefits, challenges and additional features.

#### Benefits of using the chatbot

Four main categories emerged from the analysis. These included the convenience and accessibility of the chatbot as a mental health service provision tool, the perceived affective attitudes towards the chatbot, perceived privacy and anonymity of the chatbot and the trustworthiness of the bot.

##### Convenience and accessibility.

For most participants, a major benefit of the chatbot was that it could provide mental health support conveniently and at a time and place that was of their choice. This convenience meant that accessing mental health support could easily fit into someone’s day rather than requiring planning and incurring costs (both economic and time), as one participant remarked:

Yes, it is just simple to be with it [chatbot] than to go up and down [to a clinic]. I’m sitting at home making tea and I’m busy with it [chatbot] so everything is easy and simple than to go out in the morning and come back later on. So, I just do everything while I’m at home.

—MSM, 04, IDI

The convenience of using a chatbot for mental health support was closely linked to the issue of accessibility. Being able to use the chatbot anywhere and at any time without having to make and get to appointments meant being able to access support more immediately and urgently:

…if there is such an app, [its] like, immediate, you talk to someone and say I’m thinking of killing myself and they will ask you why and you work through solutions and stuff and a lot of other things. So, it can be like an immediate response to whatever challenge you’re facing—well emotionally of course and psychologically and mentally…

—MSM, 02, IDI

##### Affective attitudes.

A major theme that was identified related to how participants felt towards their interaction with the chatbot. Overall, participants felt positively towards the chatbot, feeling that it was friendly, and enjoyed using it, noting that it was ‘like talking to a friend’ (TGW, 03, IDI) or having a casual conversation with a person. Additionally, feeling comfortable using the chatbot was an important subtheme, with most participants reporting feeling comfortable using it. This comfort was often mediated through the feelings of privacy and anonymity (see theme) participants felt they had when using the chatbot. However, comfort using a chatbot needs to be carefully monitored, our findings suggest it relies on maintaining and ensuring privacy in conversations and its ability to emotionally connect and build rapport with users:

Uhm not quite much [feeling comfortable], reason being is because the chatbot is robotic, alright the responses are automated in such a way that it doesn’t give you [emotional] responses. It almost feels like uhm they [responses] don’t relate.

—TGW, 03, IDI

Some participants also reported that a chatbot provided a means of getting mental health support without being judged for needing it:

It’s private…I don’t have to worry about people judging me for seeking help.

— TGW,01, FGD

Our data highlight two important novel benefits that improve its ability to engage users. While this is a minor finding, it provides insights into how technology could increase mental health support uptake, in particular with younger users. This minor subtheme includes the feeling that a chatbot is a modern and advanced way of accessing services, as illustrate in the following quote:

Facilitator: Okay. Also how do you think using the chatbot for mental health support, will influence how others perceive you?

Participant: I think the perception would be advancement. I am advanced instead of paying a psychologist, I have it right at the tab of my hands, yeah.

—TGW, 03, IDI

It also links to hedonic motivation (fun)^21^ to access mental health services.

Participant: Yes, it was fun and interesting. It was fun and interesting because I can ask without being afraid of judgment and to be open about everything that I am talking about with the chatbot. Yes, it was fun. It was fun, yes.

— TGW, 04, IDI

This sub-theme requires further research.

Privacy and anonymity was a major theme and was an important benefit of using a chatbot for most participants:

Participant: What might boost my confidence is knowing that nobody else has access to the chats, my secrets are safe, my emotions, stress. What I’m going through, uhm petty or not, is safe, yeah.

— TGW, 03, IDI

The chatbot also provided the benefit of feeling anonymous to some users, allowing them to be more truthful:

Participant: …I love how…I get to disclose the information, very confidential information, with someone I do not see. I think for a lot of people it’s much…easier to disclose to someone or something you do not see physically because it becomes much…easier to confide in something or someone that, that you cannot see with your own eyes rather than speaking face to face.

— MSM, 03, IDI

The benefit of feeling more anonymous could be an important benefit for many users who feel intimidated to access mental health support services in person, and who may feel nervous to disclose sensitive information in person.

##### Trust.

An important theme that overlapped with the themes of privacy and anonymity was trust. Trust in the chatbot stretched across several areas, and participants felt it would keep their information private and because it was on their phone and could be kept locked. In addition, trust came from the perceived knowledge that the chatbot had from its association with the organisations that developed it and its perceived response accuracy:

Participant: I think I would trust the accuracy pretty much 100%. Yeah, because I feel like within terms of the information, you get accurate information, accurate advice…

—MSM, 03, IDI

Trust in the chatbot was not absolute across participants however, with some participants highlighting that the chatbot is not qualified to provide mental health support—negatively impacting the acceptability of the chatbot, as highlighted below:

Participant: It’s not qualified to be a psychologist or counsellor.

Facilitator: So you don’t trust the chatbot because you are not sure if it’s qualified, you want to get the right story?

Participant: Hmm [Yes].

— TGW, 05, IDI

Trust was further mediated through beliefs about data privacy and beliefs about its ability to give accurate and quick responses that was often undermined by latency issues in the pilot chatbot. The challenges associated with the chatbot and the negative impact on acceptability are discussed in the next section.

#### Challenges with using the chatbot

Four main categories regarding challenges emerged from the analysis. These included the importance of relationship building for supporting use, poor performance, data privacy concerns and the potential or perceived costs of using the chatbot.

##### Relationship building.

Many participants reported a preference for in-person mental health counselling—either as an overall preference or as a process that would follow the use of the chatbot via referral. A major benefit of in-person counselling over the chatbot was the value add of a perceived connection between people:

I believe in talking to a person. I want to see your face; I want to see your expression. You know. If I am talking to you now, are you going to see me, are you going to see my facial expressions, is it going to feel how I feel? You know, is it going to feel the pain that I am feeling? So, sometimes, it’s great.

— TGW, 01, FGD

Building a relationship was also impacted by its perceived compromised ability to express feelings:

Participant: The problem is it doesn’t have feelings.

Facilitator: So that is a problem for you?

Participant: I can’t make a relationship with it.

—TGW, 05, IDI

Most participants were open to building a relationship with a chatbot, and more time spent on better rapport building is an important step to improving engagement and acceptability:

Participant: Yes, I think the chatbot was empathetic, because when I addressed the problem, it said, wow, you must be dealing with a lot right now. It’s sort of like building a rapport between me and the chatbot…

—TGW, 04, IDI

Participant: …another thing if the bot could be more in touch with emotions, as in show more empathy would be great…

—TGW, 03, IDI

Rapport building and spending time on building a connection is an important element for designing engaging digital services.

##### Poor performance.

User acceptability of the chatbot was negatively impacted by performance issues. Repetitive responses, slow responses, being asked too many questions and responses perceived as non-specific or lacking the appropriate or expected feedback all served to undermine the acceptability of the chatbot. The negative impact of performance issues are illustrated in the quotes below:

Slow response: ‘So, its time. In the beginning it responded but it started to take long to respond. And it started doing it slowly, taking time. Like it hasn’t responded until now. And it is off now’.

—MSM, 01, FGD

Non-specific responses: ‘Like, I was still like getting to know it…but it’s also still giving me the same response, its releasing the same messages and it’s not responding to my concern when I say the things I say it does not respond to what I am saying exactly’.

—TGW, 02, IDI

Repetitive responses: ‘Well, for me, I don’t know what others think, but whatever I put on the table for the chat bot, it kept on asking the same questions over and over again. Even when you give them the answer, it goes back to the previously asked questions’.

—TGW, 02, FGD

Multiple questions: ‘It was okay and then I mean I struggled with it not like sending immediately questions and they would ask like three major questions at once yeah, so it will be nice to like, respond, like with one thing’.

—MSM, 02, IDI

These performance issues all have a negative impact on the acceptability of the chatbot and have the secondary consequences of undermining the value of it for supporting mental health.

##### Data privacy.

The theme of data privacy straddled both the benefits and challenges of using the chatbot among MSM and TGW users. Data privacy concerns were often spoken about negatively and positively with the same participant—highlighting the complexity of beliefs around data privacy issues. This complexity is highlighted in the following exchange:

Facilitator: …What makes you comfortable to open up to it [the chatbot] [is that] it doesn’t want [your] details?

Participant: Yes, that’s also why.

Facilitator: Let’s say it wants details; would you give it?

Participant: No, it wouldn’t dare, who controls it? What kind of thing is it? My details? You see that now? Who will be controlling it?

Facilitator: So, this is a very interesting conversation. So, you are saying it’s alright if it doesn’t want your name your details but if it wants your name and details then you are worried?

Participant: Yes

— MSM, 04, IDI

##### Cost

Cost to use the chatbot was an important issue and was raised by almost all participants. High data costs are a major potential perceived problem for digital interventions in low-resource settings, where they become a reason for potential users not to use the service. As one participant said:

Yes [to question on barriers to using the chatbot], I think because it’s data consuming, because…you have to have data. If you don’t have data, you cannot access the chatbot.

—TGW, 04, IDI

Even if data costs can be minimised, they are unlikely to be eliminated. This perceived cost, especially if perceived as high from the reference point of the user, may negatively impact acceptability.

#### Additional features to improve the chatbot: horizontal scaled features to enhance service

The analysis highlighted multiple suggestions for improving the feature offering of the chatbot to improve its acceptability and usability among target users. These are presented along with illustrative quotes in Table 4, but highlights include:

- Participants emphasised the need for multiplatform access, especially via widely used apps like WhatsApp. Suggestions also included data-free usage; voice-based interactions for easier, more fluent engagements and to make it more accessible to disabled or low-literate users; and peer-support options through group chats or forums.
- Enhancing the chatbot’s ability to detect and respond to emotional cues through improved natural language processing, voice recognition or emotion recognition through real-time sentiment analysis was seen as vital for rapport building. Participants also desired continuity across sessions, such as the ability for the chatbot to remember previous chats and initiate follow-ups, mimicking more human-like relationships.
- Multilingual capabilities were deemed essential, particularly for users more comfortable expressing themselves in local languages like isiZulu.
- Strengthened data protection, anonymous user profiles and features allowing users to clear stored conversations were recommended to enhance trust and address concerns around surveillance or data misuse.
- Participants wanted the chatbot to act as a gateway to further services, including referrals to social workers, mental health professionals or emergency contacts. This layered approach could increase user safety and extend the chatbot’s utility beyond self-management.
- Some personalisation of the chatbot was deemed important by some. Suggestions included customisable avatars and voices, visually engaging interfaces and logical conversation flows that address one issue at a time. Personal touches such as automated daily greetings or progress tracking were seen as features that could enhance emotional engagement.

**Table 4.** Recommendations to improve the features of an AI-driven chatbot providing mental health support to MSM and TGWs.

Theme	Details	Quote
Accessibility and inclusivity	Chatbot should be available on multiple platforms (e.g. WhatsApp, not just Telegram)	It would be nice to have it as a web or a tab, Facebook everywhere if it’s on the web it’s the best way…cause I’m not on Telegram. I had an account long ago so if it is on the web it is perfect, very easy to access at any time you just click, and it takes you through. — AI-M-05
	Data-free access is essential	Oh, if AI can be able to respond with or without data. — AI-T-05
	Support for group discussions and peer support	A support group…we can create different…groups, like on WhatsApp you can create channels as a referral system and then there you will meet…people who are going through the same things you are going through, you can sort of…create…a timeline to say at this specific time the group chat will start and we will start having a conversation…like an online support group. — MSM-02
	Multimodal communication: voice calls, voice notes, follow-up texts	Voice notes can be fine as well. If one can’t read English, they can use the voice note: I do not know if you can do calls, the robot would talk to them via calls. —AI-T-01 So, I think that there needs to be a voice [note] for people who can’t see… — AI- MSM-01 I would like for it to send me a good morning text, because we do not get good morning texts, the chatbot could do that. — AI-T-01
Emotional intelligence and empathy	Ability to show emotional reactions and human-like connections	Uhm another thing if the bot could be more in touch with emotions, as in show more empathy that would be great… — AI-T-03
	The chatbot should provide detailed, in-depth and non-clinical responses and be able to detect and appropriately respond to emotional cues	Maybe if it had a face reader, if you take a picture then it tells you you’re not feeling OK. It will be very much helpful, maybe if it can read your voice if it’s not sounding [right] like maybe you’re crying…, and then it picks that up. If somehow it could read your emotions, how you’re feeling… — AI-T-02
	Ability to allow chatbot to remember previous chats to improve counselling	I mean [it has] to continuously give updates and send messages like it…almost becomes your friend…and then maybe on [a] specific time or specific day it automatically says ‘hey, how are you?’ And you say hey I’m okay…I know that will be difficult because it will be like the App has to remember the last conversation it had with you… — AI-M-02
Language and cultural sensitivity	Must understand and operate in multiple local languages (e.g. isiZulu, isiXhosa)	Sometimes it becomes difficult to express what you want to say in English. You’ll find that you can’t put the grammar in the correct way, but you know you can say it better in your language; isiZulu. If they could make it that it be English, and any other preferable language, it would work like a charm. — AI-M-01
Privacy and security	Strong data protection measures	…it must have protection. Because there are people who can hack this thing and act like the bot and you think you are talking with the chatbot while it’s not, its [a person who hacked your profile]… — AI-MSM-04
	Ability to create anonymous user profiles	[Facilitator: Er, yah so you like that it’s doesn’t have like identifiers that this is who from where?] Participant: No date of birth or ID number you see. — AI-T-05
Professional and emergency support	Connect users to emergency services Enable referral to professional consultants (e.g. social workers, psychologists)	Like when you say I am sick and where is the nearest clinic I can go to and then it can help you with the clinic…it searches for [available clinics nearby]. — AI-T-02 Maybe if it’s something deep it can refer you to a consultant or something like that. — AI-T-05 I feel like we need to have more requests [on the bot] that instead of it being able to call you, you must be able to send a message to the police if you feel like you are in a situation where a person is abusing you… — AI-T-FGD-01
	Option to clear your stored data after interaction	For security, as we said, to avoid being hacked, we should know where the server is, and you should have the option of clearing all of your data… —AI-M-FGD-02
User experience and design	Importance of rapport building and ensuring a visually appealing, user personalised experience (e.g. colourful interface upon opening)	I would like that it introduces itself but I would like to choose for myself the type of a person I am maybe, it should open some options, …maybe a picture of a cartoon, maybe the name, like I need to choose there, like if I want to choose a specific voice, …we have Chris, John and Emma. Like you listen to the voice notes, the examples like ‘hi I am Emma’ or something and you choose, you say okay I like this voice because it suits this person who pops out. And to add on that when it introduces, after you made your choice, like in WhatsApp we have noticed that, if you are a WhatsApp person there are some reactions and what not. I want that if you chose Emma, and we talk to Emma, she is going to react with a sticker that shows her Emma laughing. If I chose Chris initially then it reacts with stickers that match Chris smiling, crying almost like. — AI-M-FGD-01 I feel like the background be customizable, like you can change it to whatever you like, you can do a picture or some cute pictures it must give us some options that are available… — AI_M-FGD-01
	Efficient and timely responses	And respond instantly please. The repetition of questions, I try to just avoid or shy away from that. Yes, I do understand that it is follow up questions but let it not be question after question where you ask me [the same thing] Maybe try to suggest [tips to help]. — AI-M-05
	Provide options for different mental health counselling	P3: There are many mental health issues that we have. Let’s say you can choose. They are different. Let’s say you have relationship problems [bot] can lead you [to those options]. — AI-M-FGD-02
	Clear and focused responses: one issue at a time with logical flow	And also flow in terms of it listing my problems or issues that I’m facing, it should be able to tackle [them] uhm in a sequence… — AI-T-03

Overall, our findings align closely with key components of the UTAUT and the TFA. The perceived convenience, accessibility and immediacy of the chatbot reflect strong performance and effort expectancy (UTAUT) and reduced burden and opportunity cost (TFA). Positive affective attitudes—such as enjoyment and comfort—map to hedonic motivation and affective attitude, while privacy, anonymity and trust link to facilitating conditions, trust and ethicality. Conversely, performance issues, perceived lack of empathy and data costs reduce perceived effectiveness and increase burden. Collectively, these alignments suggest that acceptability and the use of chatbots for mental health support in TGW and MSM are shaped by both functional (performance, accessibility) and affective (trust, comfort, ethicality) dimensions consistent with these theoretical models.

## Discussion

The findings from this qualitative study demonstrate overall good acceptability of a mental health support chatbot among MSM and TGW in South Africa. Participants consistently viewed the chatbot as a valuable supplement to existing mental health services, particularly for early engagement and ongoing emotional support. While not seen as a replacement for in-person care, the chatbot’s ability to offer private, convenient and stigma-free access to support was widely appreciated. Our findings suggest that the chatbot was highly acceptable and could harmonise well with existing mental health support: as a means of providing information, basic mental health support services, follow-up on mental well-being and as an essential point of referral to in-person, professional mental health services when urgent, serious or required.

Our findings highlighted some important benefits of using the chatbot among MSM and TGW participants. Previous research found that ease of use is an important benefit of chatbot interventions.^1,3,10,12–14^ Our findings support these results, highlighting the convenience and accessibility of the chatbot as a key benefit by making mental health services available anywhere and at any time. This easy access and convenience may serve to remove common logistical and emotional barriers to accessing mental health care, which is especially valuable in low-resource settings where access to trained professionals is limited and long travel distances or clinic wait times are common deterrents. Importantly this constant availability could make the time between deciding to seek help, service-seeking behaviours and the actual completion of service uptake almost instantaneous.^23^ Some participants highlighted novel motivators for engagement, including the perception of the chatbot as modern and advanced, and that the chatbot provided a fun experience.^21^ These factors may be particularly salient for engaging and promoting uptake among younger users and those newly considering mental health support.

Overall, participants reported positive affective attitudes towards the chatbot, congruent with research completed in other settings.^3,10–12,14^ They described it as friendly, non-judgmental and comforting, with some participants discussing their engagements as having a level of sentience or human-like qualities. These serve an important function of rapport building, an important and desired attribute that participants felt was critical to their ongoing engagement with the chatbot. When the rapport building was insufficient, participants defaulted to preferences for in-person counselling, and if not present, a lack of sufficient relationship building could undermine engagement with healthcare chatbots.^23^ Many participants emphasised the importance of building a human connection in therapeutic contexts, which was something the chatbot was not always able to timeously simulate. The inability of the chatbot to fully recognise emotional nuance or respond empathetically in real-time limited its effectiveness for users seeking more emotionally attuned interactions.

Privacy and anonymity were central to positive feelings about the chatbot, and privacy concerns have consistently been found to be a concern with digital platform engagement.^1–3,10,12,14,24^ The chatbot’s ability to provide confidential support without requiring face-to-face interaction was seen as a benefit and could be a particularly important attribute for individuals reluctant to disclose emotional distress in traditional in-person clinical settings. This could be a particularly important benefit for key populations, where fear of stigma and judgement often prevent engagement with mental and other health services.^25–27^ Concerns about data privacy and concerns about who has control and access to data emerged as a double-edged theme. While many participants valued the anonymity the chatbot offered, there was hesitancy about providing personal information or engaging with a system they could not fully see or control. This complexity underlines the importance of transparency, data protection measures, innovation in log-in options and user autonomy in chatbot design.

Trustworthiness was a key benefit of the chatbot, but also one easily undermined. The trustworthiness of the chatbot stemmed from its technological features (e.g. secure storage on personal devices), its affiliation with reputable health organisations and its perceived ability to give responses that were perceived as accurate and appropriate. Trust was undermined by important challenges with the chatbot; concerns about the chatbot’s lack of professional qualifications and potential data breaches highlight the need for transparent design and clear communication about the chatbot’s scope and limitations. Trust was also mediated by the performance of the chatbot, while performance also served to inform the perceived value of the chatbot as a service provision tool. Performance issues were a critical concern and highlight the importance of thorough testing and user research before implementation in interventional settings. Participants described experiences of repetitive responses, slow loading times and non-specific or irrelevant feedback—all of which undermined trust and disrupted engagement. Such technical limitations diminished the chatbot’s perceived credibility and usefulness and could lead to user disengagement.^23^ Finally, cost and data usage were raised repeatedly as barriers to access. In low-resource settings, the affordability of digital interventions is critical. Participants flagged the high cost of mobile data as a key deterrent, suggesting that text-based communication apps or free or zero-rated access would be essential for scale-up. It is important to note that even if actual data use could be minimised, participants’ perception that chatbots could consume significant data may deter use and messaging about data costs will be a critical element in planned promotion and communication about technology-based interventions.

Participants provided several practical suggestions to improve the chatbot’s design, functionality and user experience. These included multiplatform access (especially via WhatsApp), data-free functionality, voice-based and multilingual capabilities to improve accessibility and peer-support options such as moderated group chats. Participants also emphasised the importance of emotional responsiveness, proposing improvements such as sentiment detection, continuity across sessions and personalised features (e.g. customisable avatars or greetings) to foster empathy and rapport. Strengthened data protection measures and options to clear stored chats were viewed as essential for trust and ongoing engagement. These user-driven improvements reinforce and extend key constructs of the UTAUT2 and TFA, showing how perceived usefulness, hedonic motivation, trust and ethicality shape acceptability in real-world use. They also highlight the dynamic nature of decision-making^23^: improved usability, emotional connection and security will support movement from initial curiosity toward sustained engagement. Collectively, these findings suggest that acceptance and use of a chatbot for mental health support is not a single act but an evolving process shaped by users’ relational and contextual experiences.

Our study has several limitations. The sample size was small and drawn from a specific geographic area, which limits representativeness. Participants were young MSM and TGW who were already engaged in HIV prevention and treatment services at the POP INN clinic. This group may differ from individuals who are less engaged in care, who are uncomfortable or inexperienced with digital platforms or who have limited access to key population-friendly services. While most participants had some access to psychosocial support and all had used social media, we did not capture detailed data on the extent of their prior mental health care use or broader digital literacy. These characteristics mean that participants may be more comfortable discussing health issues and engaging with digital tools than some other members of their communities. However, given that this study sought to understand user needs and acceptability among likely end-users of a digital mental health support tool, the sample remains highly relevant. The insights generated from this group provide a valuable foundation for designing and refining user-centred digital interventions. Future research could extend this work by exploring experiences among MSM and TGW who are not yet engaged in HIV or mental health services, or who have limited access to digital platforms, to assess the broader applicability of these findings.

## Conclusions

This study reinforces that the acceptability of AI-driven mental health chatbots is highly contingent on emotional, cultural and contextual alignment with user expectations. Designing chatbots that are not only technically competent but also empathetic, culturally sensitive and context aware will be critical for wider adoption. In low-resource settings, issues of cost, accessibility and language must be considered from the outset. Moreover, chatbots should not be positioned as a stand-alone solution but rather as part of a layered system of care (that may extend beyond purely medical services)—one that offers immediate support while facilitating pathways to human care when needed. These findings, while derived from a specific group of MSM and TGW already engaged in HIV prevention and treatment services, provide valuable insights into how AI-driven chatbots could function within real-world mental health service provision for key populations in low-resource settings. The perspectives shared by this group—who represent both a high-need and digitally active population—offer critical guidance for tailoring and expanding chatbot interventions to other vulnerable or underserved users. In doing so, this study helps to bridge the gap highlighted in the introduction, offering early evidence of the potential usefulness, appropriateness and acceptability of chatbot-based mental health support among those most affected by HIV.

Future research should move beyond acceptability and explore the effectiveness of chatbot-based mental health support. Comparative studies examining mental health outcomes among users of AI-driven chatbots versus those accessing traditional human counselling are particularly needed. Such work would provide essential evidence on whether chatbots can meaningfully improve mental health and adherence outcomes and clarify how best to integrate them into existing HIV and mental health care systems. As LLM-based chatbots grow more sophisticated, their integration into public health strategies must be guided by rigorous evaluation, user-centred design and continuous feedback loops.

## Data Availability

The data underlying this article are available in the article.

## References

[1] Wester J, Pohl H, Hosio S et al. ‘This chatbot would never…’: perceived moral agency of mental health chatbots. Proc ACM Hum Comput Interact. 2024;8:1–28.39286336

[2] Abd-Alrazaq A A, Alajlani M, Ali N et al. Perceptions and opinions of patients about mental health chatbots: scoping review. J Med Internet Res. 2021;23(1):e17828. 33439133 10.2196/17828 PMC7840290

[3] Indah Permatasari R, Parama Artha D, Satria Wiratama B et al. Overview of chatbot usage on mental health: a scoping review. BIO Web Conf. 2024;132:05002.

[4] Stannah J, Soni N, Lam JKS et al. Trends in HIV testing, the treatment cascade, and HIV incidence among men who have sex with men in Africa: a systematic review and meta-analysis. Lancet HIV. 2023;10(8):e528–42. 37453439 10.1016/S2352-3018(23)00111-X PMC11403132

[5] Cloete A, Mabaso M, Savva H et al. The HIV care continuum for sexually active transgender women in three metropolitan municipalities in South Africa: findings from a biobehavioural survey 2018–19. Lancet HIV. 2023;10(6):e375–84. 37119825 10.1016/S2352-3018(23)00059-0

[6] UNAIDS . The urgency of now: AIDS at a crossroads. Geneva: Joint United Nations Programme on HIV/AIDS; 2024. https://crossroads.unaids.org/?_gl=1%2A1g9y7sk%2A_gcl_au%2AMTQwODk4MzczLjE3Mjk0OTM3NzM.%2A_ga%2AMTk4MzUwNDM0Ni4xNzI5NDkzNzc0%2A_ga_T7FBEZEXNC%2AMTcyOTQ5Mzc3NC4xLjEuMTcyOTQ5NDA1Ni42MC4wLjA [accessed 21 October 2024].

[7] Pienaar J, Tsope L, Mabena M et al. Risk factors for PrEP and ART medication adherence challenges in cis-gender South African men who have sex with men in Johannesburg and Pretoria. Int Health. 2025;17(4):509–16. 39945576 10.1093/inthealth/ihae090 PMC12212232

[8] Godfrey C, Nkengasong J. Prioritizing mental health in the HIV/AIDS response in Africa. N Engl J Med. 2023;389(7):581–3. 37578071 10.1056/NEJMp2305399

[9] Operario D, Sun S, Bermudez A N et al. Integrating HIV and mental health interventions to address a global syndemic among men who have sex with men. Lancet HIV. 2022;9(8):e574–84. 35750058 10.1016/S2352-3018(22)00076-5 PMC7613577

[10] Haque MDR, Rubya S. An overview of chatbot-based mobile mental health apps: insights from app description and user reviews. JMIR Mhealth Uhealth. 2023;11:e44838. 37213181 10.2196/44838 PMC10242473

[11] Bakoyiannis I . Therabot for the treatment of mental disorders. Nat Ment Health. 2025;3:485.

[12] Siddals S, Torous J, Coxon A. “It happened to be the perfect thing”: experiences of generative AI chatbots for mental health. Npj Ment Health Res. 2024;3(1):48. 39465310 10.1038/s44184-024-00097-4 PMC11514308

[13] Koulouri T, Macredie R D, Olakitan D. Chatbots to support young adults’ mental health: an exploratory study of acceptability. ACM Trans Interact Intell Syst. 2022;12(2):1–39.

[14] Casu M, Triscari S, Battiato S et al. AI chatbots for mental health: a scoping review of effectiveness, feasibility, and applications. Appl Sci. 2024;14(13):5889.

[15] Dambi J, Norman C, Doukani A et al. A digital mental health intervention (Inuka) for common mental health disorders in Zimbabwean adults in response to the COVID-19 pandemic: feasibility and acceptability pilot study. JMIR Ment Health. 2022;9(10):e37968. 35960595 10.2196/37968 PMC9555820

[16] Doukani A, van Dalen R, Valev H et al. A community health volunteer delivered problem-solving therapy mobile application based on the Friendship Bench ‘Inuka Coaching’ in Kenya: a pilot cohort study. Glob Ment Health (Camb). 2021;8:e9. 34026239 10.1017/gmh.2021.3 PMC8127638

[17] Chibanda D, Cowan F, Verhey R et al. Lay health workers’ experience of delivering a problem solving therapy intervention for common mental disorders among people living with HIV: a qualitative study from Zimbabwe. Community Ment Health J. 2016;53(2):143–53. 27221123 10.1007/s10597-016-0018-2

[18] Chibanda D, Weiss H A, Verhey R et al. Effect of a primary care–based psychological intervention on symptoms of common mental disorders in Zimbabwe: a randomized clinical trial. JAMA. 2016;316(24):2618–26. 28027368 10.1001/jama.2016.19102

[19] Lewis L, Kharsany ABM, Humphries H et al. HIV incidence and associated risk factors in adolescent girls and young women in South Africa: a population-based cohort study. PLoS One. 2022;17(12):e0279289. 36542645 10.1371/journal.pone.0279289 PMC9770356

[20] Marikyan D, Papagiannidis S. Unified theory of acceptance and use of technology: a review. Available from: https://open.ncl.ac.uk

[21] Sekhon M, Cartwright M, Francis J J. Acceptability of healthcare interventions: an overview of reviews and development of a theoretical framework. BMC Health Serv Res. 2017;17(1):88. 28126032 10.1186/s12913-017-2031-8 PMC5267473

[22] Braun V, Clarke V, Braun V et al. Using thematic analysis in psychology. Qual Res Psychol. 2006;3(2):77–101.

[23] Humphries H, Knight L, van Heerdan, A. The HIV prevention decision-making cascade: integrating behavioural insights into HIV prevention efforts. Prev Med Rep. 2024;46:102870. 39257879 10.1016/j.pmedr.2024.102870 PMC11384964

[24] Audere . Artificial intelligence to enhance HIV prevention in age of disruptions. Available from: https://www.auderenow.org/what-were-thinking-about/ai-for-hiv-prevention (accessed 27 May 2025).

[25] Golub S A, Gamarel K E, Rendina H J et al. From efficacy to effectiveness: facilitators and barriers to PrEP acceptability and motivations for adherence among MSM and transgender women in New York City. AIDS Patient Care STDs. 2013;27(4):248–54. 23565928 10.1089/apc.2012.0419 PMC3624632

[26] Sullivan P S, Grey J A, Simon Rosser B R. Emerging technologies for HIV prevention for MSM: what we have learned, and ways forward. J Acquir Immune Defic Syndr (1988). 2013;63(Suppl 1):S102–7. 10.1097/QAI.0b013e3182949e85 PMC3670990 23673879

[27] Pillay D, Stankevitz K, Lanham M et al. Factors influencing uptake, continuation, and discontinuation of oral PrEP among clients at sex worker and MSM facilities in South Africa. PLoS One. 2020;15(4):e0228620. 32352969 10.1371/journal.pone.0228620 PMC7192496

